# Gene Ontology annotation of the rice blast fungus, *Magnaporthe oryzae*

**DOI:** 10.1186/1471-2180-9-S1-S8

**Published:** 2009-02-19

**Authors:** Shaowu Meng, Douglas E Brown, Daniel J Ebbole, Trudy Torto-Alalibo, Yeon Yee Oh, Jixin Deng, Thomas K Mitchell, Ralph A Dean

**Affiliations:** 1 Fungal Genomics Laboratory, Center for Integrated Fungal Research, North Carolina State University, Raleigh NC 27695, USA; 2 Current address: Lineberger Comprehensive Cancer Center, School of Medicine, CB# 7295, University of North Carolina at Chapel Hill, Chapel Hill, NC 27599-7295, USA; 3 Department of Plant Pathology & Microbiology, Texas A&M University, College Station, Texas 77843, USA; 4 Virginia Bioinformatics Institute, Virginia Polytechnic and State University, Blacksburg, VA 24061, USA; 5 Current address: Human Genome Sequencing Center, N1619 Alkek Building, One Baylor Plaza, Houston, Texas 77030, USA; 6 Current address: Department of Plant Pathology, The Ohio State University, 2021 Coffey Road, Columbus, Ohio 43210-1087, USA

## Abstract

**Background:**

*Magnaporthe oryzae*, the causal agent of blast disease of rice, is the most destructive disease of rice worldwide. The genome of this fungal pathogen has been sequenced and an automated annotation has recently been updated to Version 6 . However, a comprehensive manual curation remains to be performed. Gene Ontology (GO) annotation is a valuable means of assigning functional information using standardized vocabulary. We report an overview of the GO annotation for Version 5 of *M. oryzae *genome assembly.

**Methods:**

A similarity-based (i.e., computational) GO annotation with manual review was conducted, which was then integrated with a literature-based GO annotation with computational assistance. For similarity-based GO annotation a stringent reciprocal best hits method was used to identify similarity between predicted proteins of *M. oryzae *and GO proteins from multiple organisms with published associations to GO terms. Significant alignment pairs were manually reviewed. Functional assignments were further cross-validated with manually reviewed data, conserved domains, or data determined by wet lab experiments. Additionally, biological appropriateness of the functional assignments was manually checked.

**Results:**

In total, 6,286 proteins received GO term assignment via the homology-based annotation, including 2,870 hypothetical proteins. Literature-based experimental evidence, such as microarray, MPSS, T-DNA insertion mutation, or gene knockout mutation, resulted in 2,810 proteins being annotated with GO terms. Of these, 1,673 proteins were annotated with new terms developed for Plant-Associated Microbe Gene Ontology (PAMGO). In addition, 67 experiment-determined secreted proteins were annotated with PAMGO terms. Integration of the two data sets resulted in 7,412 proteins (57%) being annotated with 1,957 distinct and specific GO terms. Unannotated proteins were assigned to the 3 root terms. The Version 5 GO annotation is publically queryable via the GO site . Additionally, the genome of *M. oryzae *is constantly being refined and updated as new information is incorporated. For the latest GO annotation of Version 6 genome, please visit our website . The preliminary GO annotation of Version 6 genome is placed at a local MySql database that is publically queryable via a user-friendly interface Adhoc Query System.

**Conclusion:**

Our analysis provides comprehensive and robust GO annotations of the *M. oryzae *genome assemblies that will be solid foundations for further functional interrogation of *M. oryzae*.

## Introduction

*Magnaporthe oryzae*, the rice blast fungus, infects rice and other agriculturally important cereals, such as wheat, rye and barley. The pathogen is found throughout the world and each year is estimated to destroy enough rice to feed more than 60 million people [1]. A comprehensive understanding of the genetic makeup of the rice blast fungus is crucial in efforts to understand the biology and develop effective disease management strategies of this destructive pathogen.

The rice blast fungus has been the focus of intense investigation and a number of genomic resources have been generated. These include a genome sequence [2], genome-wide expression from microarray [3] and massive parallel signature sequencing (MPSS) [4], as well as large bank of T-DNA insertion mutants [5,6]. In addition, numerous genes have been functionally characterized by targeted knockout [7-18]. While these resources are of tremendous utility, much of the genome remains unexplored. Till now, only automated annotations of the predicted genes have been available. In order to maximize the utility of the genome sequence, we have developed manual curations of the predicted genes.

Providing functionality through careful and comprehensive application of a standardized vocabulary, such as the Gene Ontology (GO) requires manual curation. The GO has evolved into a reliable and rapid means of assigning functional information [19-22]. There are two types of GO annotations. One is referred to as similarity-based GO annotation, and the other is literature-based GO annotation. Similarity-based GO annotation applies computational approaches to match characterized gene products and their associated GO terms to gene products under study. Orthology-based GO annotation is a more stringent application of similarity-based GO annotation. Literature-based GO annotation involves reviewing published work and then manually assigning GO terms to characterized gene products. Currently, similarity-based GO annotation predominates since it is rapid and relatively inexpensive [21,23]. On the other hand, although literature-based annotation is time consuming, it is considered more reliable and provides a mechanism to assign previously unassigned GO terms or newly developed GO terms to proteins. Here, we present an overview of the strategy and results obtained from the integration of both approaches to assign GO terms to Versions 5 of *M. oryzae *genome.

## Methods

### *M. oryzae *genome sequence

This paper summarizes manual annotation of Version 5 of the *M. oryzae *genome sequence. At the time of submission of this manuscript, Version 6 of the genome assembly of *M. oryzae *was released by the Broad Institute. Version 6 will be annotated using the same methodology described here. A preliminary GO annotation of the Version 6 genome sequence, based on the Version 5 annotation, has been placed at our local MySQL database at .

### Sequence similarity-based GO annotation

#### Step 1

Predicted proteins of Version 5 of the *M. oryzae *genome sequence were downloaded from the Broad Institute at . GO-annotated proteins were downloaded from the Gene Ontology (GO) database at . These GO-annotated proteins were from about 50 organisms with published association with GO terms. Only three of the 50 organisms are fungi. They are *Candida albicans*, *Saccharomyces cerevisiae*, and *Schizosaccharomyces pombe*. Other organisms are from bacteria, plants, or animals etc. Proteins of these non-fungal organisms were retained to increase the number of proteins with validated functions available for matching to *M. oryzae*.

#### Step 2

Possible ortholog pairs between GO proteins and predicted proteins from *M. oryzae *genome sequence Version 5 were estimated by searching for reciprocal best hits using BLASTP (e-value < 10^-3^) [24].

#### Step 3

Significant alignment pairs with 80% or better coverage of both query and subject sequences, 10^-20 ^or less BLASTP E-value, and 40% or higher of amino acid identity (pid) were manually reviewed.

#### Step 4

The functions of significantly matched GO proteins were manually cross- validated using data from wet lab experiments, and the NCBI Conserved Domain Database (CDD) [25].

#### Step 5

If the functions suggested from different sources were consistent with each other, and with available *M. oryzae *data, the functions of the experimentally characterized, significantly matched GO proteins, were transferred to the *M. oryzae *proteins in our study, and given the evidence code ISS (Inferred from Sequence Similarity) [26,27].

#### Step 5

The information was recorded into a gene association file following the format standard at .

### Literature-based GO annotation

#### Step 1

Literature at public databases such as PubMed [a database of biomedical literature citations and abstracts at the National Center for Biotechnology Information (NCBI)] were searched using key words, including alternative species names for the organism such as *Magnaporthe grisea *and *Pyricularia oryzae*.

#### Step 2

Relevant published papers were read and genes or gene products and their functions were identified.

#### Step 3

Where necessary, gene IDs and sequences at public databases, such as the NCBI protein database were identified.

#### Step 4

Based on the functions identified in the paper(s), appropriate GO terms were found using AmiGO, the GO-supported tool for searching and browsing the Gene Ontology database.

#### Step 5

Evidence codes were assigned following the guide at .

#### Step 6

Data were recorded into the gene association file manually or using custom PERL scripts for large gene sets with the same biological process.

### Integration of the results from the two types of GO annotations

#### Step 1

Similarity-based annotations were replaced with literature-based annotations, where redundant, using custom PERL scripts.

#### Step 2

Custom PERL scripts were used to annotate each protein with GO terms from the three ontologies using the following protocol. Any protein not annotated with a GO term following similarity-based and literature-based GO annotations was annotated with the three root GO terms, GO:0005575 (Cellular Component), GO:0003674 (Molecular Function), and GO:0008150 (Biological Process). Additionally, if any protein was lacking annotation from any of the three GO categories, Cellular Component, Molecular Function, or Biological Process, the protein was annotated with the root GO terms of the missing GO categories.

#### Step 3

Errors in the gene association file were checked using the script, filter-gene-association.pl, which was downloaded from the GO database at .

The gene association file for Version 5 of the *M. oryzae *genome sequence was uploaded to the GO database at . Many protocols and scripts were created for generating and parsing the data. For example, a protocol and five scripts were developed to replace redundant similarity-based annotation with literature-based annotation. Furthermore, a protocol and eight scripts were developed to provide each gene with a GO term from the three ontologies. In addition, a PERL script to record many genes into the gene association file was developed. This script, with slight modification, easily recorded different types of data, such as microarray expression, MPSS, or T-DNA insertion mutation, etc., into the gene association file. These protocols and scripts are available upon request from the corresponding or the first author.

## Results

### Computational GO annotation

**Figure 1:**
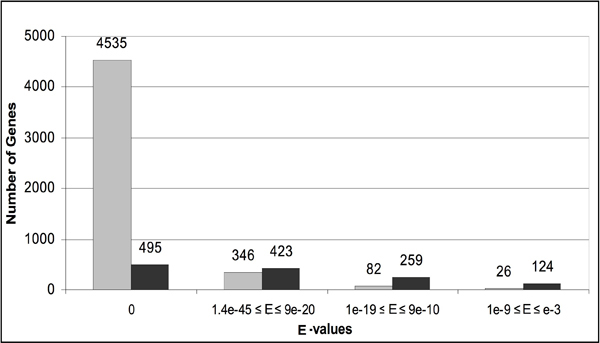
**Features of reciprocal best BLASTP matches between GO-annotated proteins and predicted proteins of *Magnaporthe oryzae***. The vast majority of the matches to characterized proteins have high sequence identity over much of their length. Shaded grey bars indicate matches with a percentage of identity (pid) ≥ 40%, and shaded black bars indicate pid < 40%.

From the initial BLASTP analysis for reciprocal best hits, 6,286 (49% of the 12,832) predicted proteins were annotated with 1,911 distinct and specific GO terms out of a total of 29,126 assigned terms. Totally, 4,881 (78%) of the 6,286 proteins were considered to be significant matches to characterized GO proteins, with an E-value < 10^-20 ^and percentage of identity (pid) ≥ 40%. Furthermore, 4,535 (93%) of the 4,881 proteins were annotated based on highly significant similarities with E-values = 0 and pid ≥ 40% (see Figure 1 for details). The pairwise alignments of these significant matches were manually reviewed. Additionally, these high quality matches were cross-validated as follows:

A total of 67 secreted proteins of *M. oryzae *was experimentally demonstrated to be secreted through cloning into an overexpression vector and expressed in *M. oryzae *transformants (Ebbole and Dean, unpublished data). These 67 secreted proteins were annotated with a biological process term GO:0009306 ("protein secretion") and a cellular component term GO:0005576 (extracellular region). An evidence code IDA was assigned to annotations of these 67 proteins since function was determined through direct assay.

A total of 128 curated cytochrome P450's of *M. oryzae *were validated by comparison and analysis of gene location and structure, clustering of genes, and phylogenetic reconstruction [28]. Different subsets of these proteins were annotated with different GO terms. For example, 75 of the 128 P450 proteins were annotated with the molecular function term GO:0005506 ("iron ion binding"), and 40 P450 proteins with the molecular function term GO:0016491 ("oxidoreductase activity"). An evidence code IGC was assigned to annotations of these P450 proteins since annotations were based on genomic context.

A total of 428 putative transcription factors of *M. oryzae *were validated by integrated computational analysis of whole genome microarray expression data, and matches to InterPro, pfam, and COG [3]. Again, different subsets of the 428 proteins were annotated with different GO terms. For example, 36 proteins were annotated with GO:0005975 ("carbohydrate metabolic process"), and 12 proteins were annotated with GO:0006520 ("amino acid metabolic process"). An evidence code RCA was assigned to annotations of the 428 transcription factors since the annotations were based on reviewed computational analysis.

A total of 2,548 conserved domains from NCBI CDD were used as evidence for cross-checking putative functions, but no GO annotation was made based solely on identification of these domains.

In addition, the evidence code ISS was assigned to annotations of 216 *M. oryzae *proteins for the following reasons: 1) These proteins have significant similarity to experimentally-characterized homologs over the majority (at least 80%) of the full length sequences. 2) The pairwise alignments of good matches between the characterized proteins and the proteins of *M. oryzae *were manually reviewed. 3) Functional domains were conserved between the *M. oryzae *proteins and their homologs. 4) The GO assignments from the characterized match proteins to the *M. oryzae *proteins were manually determined to be biologically relevant.

The remaining 1,343 proteins with a reciprocal BLASTP best match of e-value > 10^-20 ^and pid < 40% were assigned GO terms from their characterized matches, but the evidence codes were identified as IEA (Inferred from Electronic Annotation).

In sum, GO terms were assigned to 6,286 proteins of *M. oryzae*. Among the 6,286 proteins, 2,732 hypothetical proteins, 125 predicted proteins, and 14 unknown proteins were assigned functions.

### Literature-based GO annotation

More than 400 research articles were read, and 71 genes with gene knockout mutations and with accession numbers and sequences deposited in public databases such as NCBI were manually annotated using GO terms, including newly developed Plant-Associated Microbe Gene Ontology (PAMGO) terms. Gene products were annotated with GO terms relevant to their biological functions. For example, 6 genes were annotated with GO:0000187 ("activation of MAPK activity"), 5 genes with GO:0075053 ("formation of symbiont penetration peg for entry into host"), 14 genes with GO:0044409 ("entry into host"), 8 genes with GO:0044412 ("growth or development of symbiont within host"), and 43 genes with GO:0009405 ("pathogenesis"). The evidence code IMP (inferred from Mutant Phenotype) was assigned to these annotations since gene-knockout mutants were generated in order to determine functions of these genes.

A total of 210 genes were annotated on the basis of published microarray studies [3]. Again, gene products were annotated with GO terms, including PAMGO terms, relevant to their biological functions. For example, 67 genes were annotated with GO:0044271 ("nitrogen compound biosynthetic process"), 27 genes with GO:0075005 ("spore germination on or near host"), 26 genes with GO:0075035 ("maturation of appressorium on or near host"), and 114 genes with GO:0075016 ("appressorium formation on or near host"). The evidence code IEP (Inferred from expression Pattern) was assigned to these annotations on the basis that the genes were up-regulated by at least 10-fold in association with the particular biological process. A further 2,433 genes were annotated on the basis of published Massively Parallel Signature Sequencing **(**MPSS) studies [4], including 1,041 genes annotated with GO:0043581 ("mycelium development"), and 1,392 genes annotated with GO:0075016 ("appressorium formation on or near host"). The evidence code IEP was also assigned to these annotations since the genes were up-regulated only during a certain biological process, such as mycelium formation, and the fold change was equal to or greater than 10.

On the basis of whole genome T-DNA insertion mutation data [5], 120 genes were annotated with relevant GO terms and PAMGO terms. For instance, 43 genes were annotated with GO:0030437 ("ascospore formation"), 14 genes with GO:0009847 ("spore germination"), 64 genes with GO:0075016 ("appressorium formation on or near host"), and 106 genes with GO:0009405 ("pathogenesis"). An evidence code IMP (inferred from mutant phenotype) was assigned to these annotations.

In total, 2,810 proteins were annotated based on experimental data from published peer-reviewed literature. Of these, 1,673 proteins were annotated with terms created by the PAMGO consortium to describe interactions between symbionts and their hosts.

### Integration of results from the two types of GO annotations

Integration of the similarity-based and literature-based annotation resulted in 7,412 proteins being annotated with specific GO terms, covering more than 57% of the inferred proteome. The remaining 5,464 predicted proteins, not having high similarity to GO-annotated proteins, were annotated with three general GO terms. GO:0005575 (Cellular Component), GO:0003674 (Molecular Function), and GO:0008150 (Biological Process). Therefore, our GO annotation provides an annotation of the entire 12,832 proteins predicted in *M. oryzae*, and each protein being annotated with GO terms from the three GO categories.

### Data availability

The GO annotation of Version 5 of the genome sequence of *Magnaporthe oryzae *is available at the GO Consortium database .

## Discussion

Here, we present a detailed protocol for integrating the results of similarity-based annotation with a literature-based annotation of the predicted proteome of Version 5 of the genome sequence of the rice blast fungus *M. oryzae*. Through careful manual inspection of these annotations, we are able to provide a reliable and robust GO annotation for more than half of the predicted gene products. Of 6,286 proteins receiving computational annotations, only 1,343 did not exceed our stringent match criteria upon manual review and so were assigned the evidence code IEA. It should be noted that annotations with the IEA evidence code are retained in the GO database for only one year, and then the GO Consortium will remove them from a gene association file. To be retained, IEA annotations must be manually reviewed in order to be assigned an upgraded evidence code such as ISS (Inferred from Sequence or Structural Similarity). Currently, there is no recognized standard to assign the ISS code. We recommend the following criteria for assigning the ISS code:

• The functions of the proteins from which the annotation will be transferred must be experimentally characterized.

• The similarity between the characterized proteins and the proteins under study must be significant. For example, we used ≥ 80% coverage of both query and subject sequences, ≤ 10^-20 ^E-value, and ≥ 40% percentage of identity (pid) as cutoff criteria in our similarity-based GO annotation. Ideally, orthology should be established by phylogenetic analysis.

• The pairwise alignment between the characterized proteins and the proteins under study should be manually reviewed and cross-validated with characterized or reviewed data of other resources such as functional domains, active sites, and sequence patterns etc.

• Biological appropriateness of all assigned GO terms should be manually reviewed.

## Competing interests

The authors declare that they have no competing interests.
